# Inhibition of Protein Farnesylation Arrests Adipogenesis and Affects PPARγ Expression and Activation in Differentiating Mesenchymal Stem Cells

**DOI:** 10.1155/2007/81654

**Published:** 2007-12-09

**Authors:** Daniel Rivas, Rahima Akter, Gustavo Duque

**Affiliations:** ^1^Lady Davis, Institute for Medical Research, Montreal, Quebec, Canada QC H3T 1E2; ^2^Nepean Clinical School, University of Sydney, Penrith, NSW 2750, Australia

## Abstract

Protein farnesylation is required for the activation of multiple proteins involved in cell differentiation and function. In white adipose tissue protein, farnesylation has shown to be essential for the successful differentiation of preadipocytes into adipocytes. We hypothesize that protein farnesylation is required for PPARγ2 expression and activation, and therefore for the differentiation of human mesenchymal stem cells (MSCs) into adipocytes. MSCs were plated and induced to differentiate into adipocytes for three weeks. Differentiating cells were treated with either an inhibitor of farnesylation (FTI-277) or vehicle alone. The effect of inhibition of farnesylation in differentiating adipocytes was determined by oil red O staining. Cell survival was quantified using MTS Formazan. Additionally, nuclear extracts were obtained and prelamin A, chaperon protein HDJ-2, PPARγ, and SREBP-1 were determined by western blot. Finally, DNA binding PPARγ activity was determined using an ELISA-based PPARγ activation quantification method. Treatment with an inhibitor of farnesylation (FTI-277) arrests adipogenesis without affecting cell survival. This effect was concomitant with lower levels of PPARγ expression and activity. Finally, accumulation of prelamin A induced an increased proportion of mature SREBP-1 which is known to affect PPARγ activity. In summary, inhibition of protein farnesylation arrests the adipogenic differentiation of MSCs and affects PPARγ expression and activity.

## 1. INTRODUCTION

A common phenomenon seen during the normal aging process is the redistribution of fat which accumulates in usually non-fat tissues [1–4]. Several hypotheses have been tested to explain age-related fat accumulation outside adipose tissue including a possible reduction in the capacity to metabolize fatty acids [5], a predominance of lipodystrophy [6], or finally a pure process of dedifferentiation of nonadipose mesenchymal stem cell (MSCs) into adipocytes-like cells [6–8].

Indeed, bone is not the exception to this phenomenon. One of the characteristics of senile osteoporosis is the predominance of adipose tissue within the bone marrow associated with a significant reduction in osteoblastogenesis and thus in bone formation [4, 9]. The predominance of adipogenesis seen in aging bone is the consequence of mesenchymal stem cells “dedifferentiation” which induces them to remain in a preadipocytic stage [7, 10].

There is evidence that among the multiple mechanisms involved in adipogenesis, protein farnesylation is essential for the differentiation of white fat precursors into mature adipocytes [11]. When human preadipocytes were induced to differentiate in the presence of insulin, addition of inhibitors of farnesylation affected their differentiation and decreased peroxisome proliferator activator gamma (PPAR*γ*) expression [11]. Therefore, it is tempting to propose that, as in white fat, protein farnesylation could be necessary for the successful differentiation of MSCs into adipocytes within the bone marrow.

Lamin A is an example of a protein that not only requires farnesylation to be activated [12] but also plays an important role in adipogenesis [13]. Lamin A belongs to the group of proteins that form the lamina which keeps the nuclear envelope playing a role in a number of nuclear processes including DNA replication and cell differentiation [12, 14]. Alterations in lamin A activation as well as mutations in the lamin A encoding gene are known as “laminopathies.” In humans, lamins have been linked to Familial partial lipodystrophy (FPLD) a disease that is characterized by adipose tissue repartitioning with multiple metabolic disturbances, including insulin resistance and dyslipidemia [15]. Lamins have also been associated with other type of lipodystrophies such as Dunnigan-type Familial partial lipodystrophy [13]. Due to the fact that all these models of lamin A mutations affect adipogenesis and in some cases PPAR*γ* expression and activity [13, 15], we hypothesize that protein farnesylation in general and lamin A farnesylation in particular could be required for adipogenesis in a model of adipogenic differentiating mesenchymal stem cells (MSCs). In summary, the determination of the potential effect that protein farnesylation has on adipogenesis and PPAR*γ* expression in the bone marrow could offer a new approach to the understanding of the pathophysiology and treatment of senile osteoporosis.

## 2. MATERIALS AND METHODS

**Reagents**

FTase inhibitor-277 (FTI-277) was purchased from Sigma-Aldrich Corporation (St. Louis, Mo, USA). FTI-277 was dissolved in Dimethyl sulphoxide and then filter-sterilized using a 0.2 *μ*m filter. Other reagents were from Sigma-Aldrich Corporation unless stated otherwise.

**In vitro Differentiation of MSCs**

Human MSCs (BioWhittaker, Walkersville, Md, USA) were induced to differentiate into adipocytes as previously described [16]. Briefly, MSCs were plated at a density of 5 × 10^5^ cells per well in 100 cm^2^ dishes containing MSCs growth media (BioWhittaker, Walkersville, Md, USA) with 10% fetal calf serum (FCS) and incubated at 37°C for 24 hours. After the cells reached 60% confluence, media were replaced with MSCs growth media or induced to differentiate into adipocytes using adipogenesis induction media (AIM) (prepared with DMEM, 4.5 g/L glucose, 1 *μ*M dexamethasone, 0.2 mM indomethacin, 1.7 *μ*M insulin, 0.5 mM 3-isobutyl-1-methylxanthine,10% FCS, 0.05 U/mL penicillin, and 0.05 *μ*g/mL streptomycin) for 3 days, incubated 3 days in adipogenesis maintenance medium (DMEM, 4.5 g/L glucose, 1.7 *μ*M insulin, 10% FBS, 0.05 U/mL penicillin, and 0.05 *μ*g/mL streptomycin), and then switched to induction media again to promote adipogenic phenotype as previously described [16]. In all experiments, media were changed every three days.

**Identification of the effect of FTI-277 on adipocyte differentiation**

MSCs were plated in 4 cm^2^ dishes in a density of 4 × 10^4^ cells per dish. At 60% confluence, media were replaced with AIM containing either FTI-277 (5–10 *μ*M) or vehicle alone. At timed intervals (weeks 1, 2, and 3), media were aspirated and cells were stained for oil red O and counterstained with hematoxylin. Differentiated adipocytes were considered those polygonal in shape, with eccentrically located nuclei, considerable cytoplasm, and lipid droplets scattered throughout.

**Identification of nuclear blebbing using Propidium Iodide Staining**

Cells were plated in 6-well plates, induced to differentiate, and treated as previously described. After 2 weeks of differentiation and treatment, cells were fixed using 70% ethanol for 20 minutes. After thorough washing in PBS, cells were stained for nuclear red fluorescence using propidium iodide. Nuclei were then observed via UV lightusing an Olympus IX-70 microscope (Olympus, London, UK). Cells showing deformities in the nuclear shape or vacuolization were considered positive for blebbing as previously described [17].

**Measurement of viable cells after treatment with FTI-277**

MSCs were seeded at a density of 4 × 10^2^ cells/well in 96-well cluster plates (Falcon, Becton-Dickinson, NJ, USA). At 60% confluence, cells were committed to differentiate into adipocytes as previously described. Cells were treated with increasing concentrations of FTI-277 (5–10 *μ*M) or with vehicle alone. Cell viability was assessed using MTS Formazan before induction (time 0) and 48–72 hours after differentiation was induced. MTS Formazan assesses mitochondrial function by the ability of viable cells to convert soluble 3-(4,5-dimethylthiazol-2-yl)-2,5-diphenyltetrazolium bromide (MTS) into an insoluble dark blue Formazan reaction product measured photometrically as previously described [18]. A stock solution of MTS was dissolved in PBS at a concentration of 5 mg/mL and was added in a 1 : 10 ratio (MTS/DMEM) to each well incubated at 37°C for 4 hours and the optical density determined at a wavelength of 570–630 nm on a microplate reader model 3550 (Biorad, Hercules, Calif, USA). In preliminary experiments, the absorbance was found to be directly proportional to the number of cells over a wide range ($2\times 10^{2}-5\times 10^{4}$ cells/well). The percent survival was defined as [(experimental_absorbance_ − blank_absorbance_)/control_absorbance_ − blank_absorbance_)] × 100, where the control_absorbance_ is the optical density obtained for 1 × 10^4^ cells/well (number of cells plated at the start of the experiment), and blank_absorbance_ is the optical density determined in wells containing medium and MTS alone.

**Western blot analysis**

MSCs were treated as previously described and then lysed in 20 mM tris-HCl, pH 7, 5, 200 mM DTT, 200 mM KCl, 0.5 ml glycerol and protease inhibitor tablets (Roche Diagnostics Canada, Laval, QC, Canada), freeze-thawed 3 times in a dry ice-ethanol bath and centrifuged at 11,500 rcf for 15 minutes to remove insoluble material. Lysates were dissolved in SDS electrophoresis buffer (Bio-Rad, Hercules, Calif, USA) and proteins separated on SDS-polyacrylamide gels and subsequently electrotransfered to polyvinylidene difluoride membranes. After membrane blocking with PBS containing 0.1% Tween 20 and 10% non-fat dry milk, membranes were incubated overnight at 4°C using an antibody directed against prelamin A (which crossreacts with lamin C), PPAR*γ*, sterol regulatory element binding protein 1 (SREBP-1), lamin B, and the chaperon protein HDJ-2 (Santa Cruz Biotechnology, Santa Cruz, Calif, USA). The bound antibodies were detected with the corresponding secondary antibodies conjugated with horseradish peroxidase (HRP). Blots were developed by enhanced chemiluminescence using Lumi-GLO reagents (Kirkegoard & Perry, Gaithensburg, Mass, USA).

**PPAR*γ* activity measurement**

DNA binding PPAR*γ* activity was determined using the ELISA-based PPAR*γ* activation TransAM kit (Active Motif, Rixensart, Belgium) as previously described [16]. The Trans-AM PPAR-Kit contains a 96-well plate on which an oligonucleotide containing a peroxisome proliferator response element (PPRE) ($5^{\prime }$-AACTAGGTCAAAGGTCA-$3^{\prime }$) has been immobilized. PPAR-contained in nuclear extract specifically binds to this oligonucleotide. The primary antibody used in the Trans-AM PPAR-Kit recognizes an accessible epitope on PPAR-protein upon DNA binding. Addition of a secondary horseradish peroxidase (HRP)-conjugated antibody provides a sensitive colorimetric readout easily quantified by spectrophotometry (450 nm). To quantify PPAR-activation, 20 *μ*g of nuclear extract was measured using the Trans-AM PPAR Kit according to the manufacturer's instructions (Active Motif, Carlsbad, Calif, USA).

**Statistical analysis**

All results are expressed as mean ± standard error of the median (SEM) of 3 replicate determinations. Statistical comparisons are based on oneway analysis of variance (ANOVA) for different time intervals or Student's t-test. A probability value of *P* < .05 was considered significant.

## 3. RESULTS AND DISCUSSION

The progression of MSCs differentiation entails the up and down regulation of multiple genes that will induce a change in cell phenotype as well as cell function [19]. This process has been widely described and involves a three-week exposure to differentiation media in which cells exposed to insulin-containing adipogenesis induction media become preadipocytes at week 2 and mature adipocytes at week 3 [20, 21]. The widely reported gene changes, occurring both in vitro [22] and in vivo [19], have provided to the field of bone research an armamentarium to potential therapeutic targets for senile osteoporosis [8, 20].

With aging, there is a predominant adipogenic differentiation of bone marrow MSCs which is mostly associated to high expression of PPAR*γ*2 [23, 24]. This factor determines the commitment of MSCs into adipocytes at the expense of their differentiation into osteoblast with a subsequent decline in bone formation [5, 8].

Overall, although there is a correlation between aging and the transcription factors for bone marrow adipogenesis [23], the link between them and the wholesome aging process remains unclear.

Protein farnesylation is an essential step required for the activation of several proteins involved in adipogenesis (i.e., GLUT-4, CREB, p21) [11]. Farnesylation is activated by a protein farnesyltransferase (FTase) which adds a 15-carbon farnesyl group to the cystein found within the Caa*X* motif [25, 26]. This addition will induce the activation of multiple proteins such as p21, HDJ-2, and lamins (A/C and B) [26]. Protein farnesylation could be inhibited using inhibitors of FTase.

In the case of fat, insulin-stimulated prenylation of the Ras family GTPases triggers the intrinsic cascade of adipogenesis [11]. This effect is inhibited by FTI-277 in subcutaneous fat cells thus affecting adipocyte differentiation of preadipocytes [11, 15]. In contrast, the effect of inhibition of protein farnesylation in human MSCs committed to differentiate into adipocytes remains unknown.

Among the proteins that require farnesylation to be activated, lamin A seems to play an important role in adipogenic differentiation of MSCs. In fact, two studies have found changes in lamin A expression in normal models of adipocyte differentiation [27, 28]. The first one identified lamin expression in human adipose cells both in relation to anatomical site and differentiation state finding that lamin A and B1, but not B2, were expressed in mature human adipocytes whereas preadipocytes expressed all four lamins [27]. A second study looked at proteomic changes in adipocyte differentiation of cells obtained from subcutaneous fat. Amongst the 170 protein features found in their study at day 9 of differentiation, lamin A expression was included in the group of proteins of the cytoskeleton with >3-fold reduction in its expression [28].

Recent evidence looking at the role of lamins in adipogenesis has demonstrated that overexpression of lamin A inhibits adipogenic differentiation of 3T3 preadipocytes [12]. This effect was associated with inhibition of expression of PPAR*γ*2. In contrast, fibroblasts obtained from mice lacking lamin A showed higher potential to differentiate into adipocytes. This evidence suggests that a reduction in lamin A expression, which may happen with aging, would facilitate the differentiation of MSCs into adipocytes.

Indeed, Young et al [25] have suggested that neither the presence nor the absence of lamin A explains by itself the physiologic role of lamin A in cell function and differentiation. They demonstrated that lamin A could be negligible without affecting cell function and differentiation [25]. Therefore, they propose that it is farnesylation and not lamin A itself that could be important for disease pathogenesis.

In fact, both the absence of lamin A and the presence of high levels of prelamin A seem to play opposing roles in adipogenesis in several models of subcutaneous fat. Total absence of lamin A would stimulate adipogenesis [12] whereas increased levels of prelamin A due to lack of farnesylation inhibit adipogenesis through to the inhibition of PPAR*γ* activity [15].

Since subcutaneous and bone marrow fat could have significant physiological differences, in this study we decided to test if inhibition of lamin A farnesylation has similar effect on human MSCs than the effect seen in subcutaneous fat.

**Figure 1:**
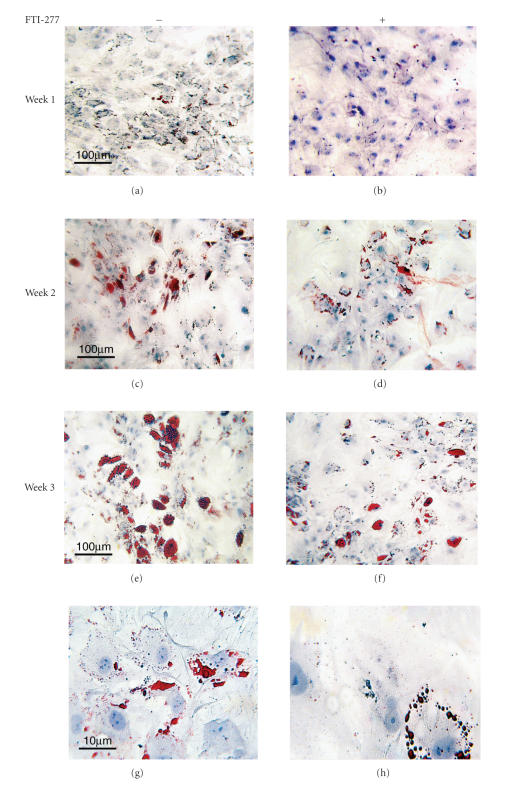
Effect of FTI-277on adipogenesis: human MSCs were committed to differentiate into adipocytes and treated for three weeks with either FTI-277 (5 *μ*M) (b, d, f, and h) or vehicle alone (a, c, e, and g). At timed intervals (week 1 (a and b), week 2 (c and d), and week 3 (e and f)), cells were fixed, stained with oil red o, and counterstained with hematoxylin to assess adipocyte differentiation. Lower magnification (10×) shows higher amount of fat droplets (red) and differentiated adipocytes in untreated cells at all time points (a, c, and e) as compared with FTI-277-treated cells (b, d, and f). At higher magnification (100×), the amount and distribution of fat droplets is highly affected by treatment (h) where lipid droplets (red) are unable to reach confluence as compared with untreated cells (g). Note the changes in the cytoplasm after treatment (h) including vacuolization, irregular nuclei, and “mega” cytoplasm.

**Figure 2:**
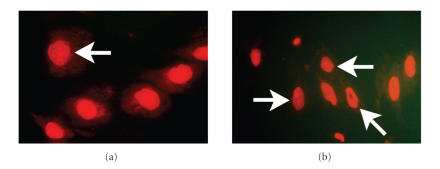
Nuclear changes in differentiating MSCs after inhibition of protein farnesylation: cells were plated and induced to differentiate as previously described. At week 2 of differentiation, cells were fixed and stained using propidium iodide to identify nuclear changes (blebbing and vacuolization). The figure shows the changes in nuclear morphology compatible with blebbing (white arrows) in most of the cells after treatment with FTI-277 (5 *μ*M) (b). In contrast, untreated cells (a) showed fewer changes compatible with blebbing. Morphologically, cells treated with FTI-277 showed smaller nuclei than AIM-treated cells. Photomicrographs were taken at ×100 magnification and represent three different experiments.

**Figure 3:**
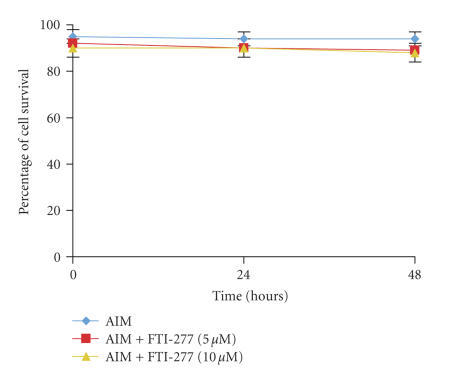
Effect of FTI-277 on survival of adipogenic differentiating MSCs: MSCs were plated 96-well plates and induced to differentiate into adipocytes. Cells were treated with either FTI-277 (5–10 *μ*M) or vehicle alone. After 24 and 48 hours, cell survival was assessed by MTS Formazan as described in methods. There was no difference between treated and nontreated cells at both time intervals. This experiment was repeated three times.

Differentiating MSCs were treated with an inhibitor of protein farnesylation and the changes in their phenotype and capacity to produce fat droplets assessed. As shown in Figure 1, cells treated with FTI-277 showed changes in their phenotype which include cytoplasm vacuolization, big nuclei, and decreased capacity to produce fat. Furthermore, in agreement with previous reports on nuclear changes induced by lack of lamin A activity, treated cells showed nuclear changes compatible with nuclear blebbing and vacuolization (see Figure 2) [18]. These changes did not have an effect of cell survival (see Figure 3).

**Figure 4:**
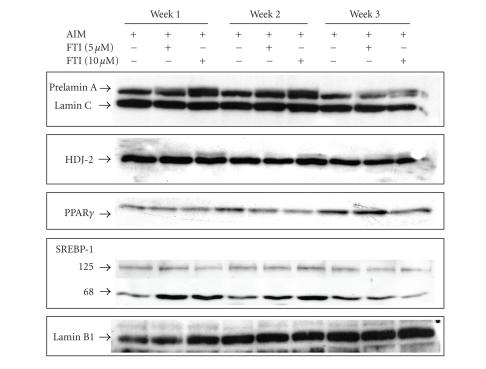
Effect of FTI-277 on protein farnesylation and transcription factors for adipogenesis in differentiating MSCs: human MSCs were plated in 6-well plates as previously described. After confluence, media were replaced with AIM with FTI-277 (5 and 10 *μ*M) or vehicle alone. Media were replaced every three days for three weeks. Nuclear extracts were obtained at weeks 1, 2, and 3 of differentiation and treated as described in Materials and Methods. Membranes were incubated overnight at 4°C using an antibody directed against either prelamin A, HDJ-2, PPAR*γ*, SREBP-1, and lamin B1. The bound antibodies were detected with the corresponding secondary antibodies conjugated with horseradish peroxidase. Blots were developed by enhanced chemiluminescence using Perkin-Elmer reagents. Treatment with increasing doses of FTI-277 induced an increase in both, prelamin A and unfarnesylated HDJ-2 expression (second upper band) suggesting that FTI-277 was effective on inhibiting farnesylation in this model of MSCs differentiation. Although a lower expression of both prelamin A and HDJ-2 at week 3 of differentiation was found, the presence of an upper band in the treated cells suggests that inhibition of farnesylation by FTI-277 was still effective. Furthermore, inhibition of farnesylation correlates with lower levels of PPAR*γ*. Finally, at weeks 1 and 2, a sharp SREBP-1 68-kDa band (mature) correlates with higher levels of prelamin A expression whereas the 125-kDa precursor proteins is much less intensely stained. These results suggest that inhibition of farnesylation affects adipogenesis due to reduced expression of PPAR*γ* which correlate with higher levels of mature SREBP-1. Membranes were stripped and immunoblotted for lamin B1 levels to demonstrate equal loading of proteins. The images are representative of three different experiments.

To test if in effect there was an inhibition in protein farnesylation, we assessed the expression of two proteins that require farnesylation to be activated, lamin A and the chaperon protein HDJ-2. These two proteins are considered as key markers of effective inhibition of farnesylation [29]. As shown in Figure 4, the presence of a double upper band demonstrates the presence of prelamin A and unfarnesylated HDJ-2 probing that FTI-277 inhibits farnesylation in this model in a dose-dependent manner. This effect was more significant at week 1 and 2 of differentiation suggesting that the effect was more significant during the preadipocyte stages. However, although there is a reduction in both HDJ-2 and prelamin A at week 3 of differentiation, the double upper band remains visible (see Figure 4).

Furthermore, we were interested in looking at the effect that inhibition of farnesylation has on PPAR*γ*2 expression and activity. A previous study using subcutaneous fat has demonstrated that accumulation of prelamin A induced a reduction in the levels of PPAR*γ* expression [15]. In agreement with their results, our study using human MSCs shows a reduction in the levels of PPAR*γ* expression (see Figure 4) at all time intervals (weeks 1, 2 and 3). Furthermore, at weeks 1 and 2, the lower expression of PPAR*γ* correlates with a significantly increased proportion of mature SREBP-1. The fact that a higher proportion of mature SREBP-1 is found in FTI-treated cells is also in agreement with previous reports which suggest that sequestration of SREBP-1 by prelamin A has an inhibitory effect on PPAR*γ* activity [15, 30]. This effect was predominantly found during the preadipocyte stages.

**Figure 5:**
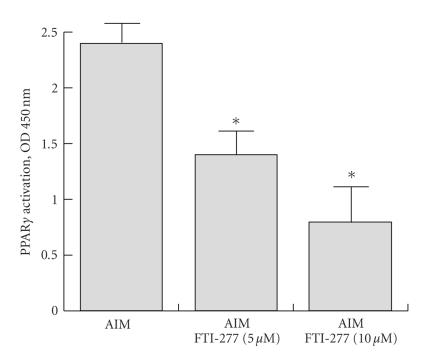
Effect of FTI-277 on PPAR*γ*2 activity: PPAR*γ* DNA binding activity was determined using ELISA-based PPAR*γ* activation kit and quantified by colorimetry. The levels of activity after treatment with either AIM or AIM + FTI-277 (5–10 *μ*M) are shown. Both dosages (5 and 10 *μ*M) significantly reduced the activity of the PPAR*γ* complex in the nuclei. Values are mean ± SEM of 6 wells per group in three independent experiments; ^*^ *P* < .01 versus matched untreated cells.

Finally, from a mechanistic approach, we looked at the PPAR*γ*2 nuclear complex activity in order to identify if protein farnesylation is required for effective activation of this complex. We found that treatment with FTI-277 affects the PPAR*γ*2 nuclear complex in a dose-dependent manner (see Figure 5).

Overall, in this model of human MSCs differentiation, we have found that inhibition of farnesylation has an effect on adipogenesis simultaneously affecting PPAR*γ*2 expression and activity more markedly during the preadipocyte stages of differentiation (week 1 and 2). A potential limitation of our study is that pharmacological inhibition of farnesylation could affect many of the proteins that are required in adipogenesis. Therefore, further studies looking at farnesyltransferase knockdown in this model should be pursued.

In summary, our results outline the role of protein farnesylation in bone marrow adipogenesis and more specifically in the activation of PPAR*γ* in a model of insulin-induced bone marrow adipogenesis.
